# Lipid distribution, composition and uptake in bovine articular cartilage studied using Raman micro‐spectrometry and confocal microscopy

**DOI:** 10.1111/joa.12624

**Published:** 2017-05-16

**Authors:** Jessica Claire Mansfield, C. Peter Winlove

**Affiliations:** ^1^ College of Engineering, Mathematics and Physical SciencesUniversity of ExeterExeterDevonUK

**Keywords:** cartilage, lipids, Raman

## Abstract

The distribution and composition of endogenous lipids in articular cartilage and transport of exogenous fatty acids have been investigated on a microscopic scale in fresh bovine articular cartilage. To investigate the distribution and composition of the endogenous lipids, hyperspectral Raman maps were taken of chondrocytes and their surrounding matrix in both the deep and superficial zones. These revealed differences in both lipid distribution and composition between the two zones. Extracellular lipid was observed surrounding the cells in the superficial zone but not in the deep zone. Additionally, intracellular lipid droplets were observed that were larger and more numerous in the deep zone (*P *= 0.01). The extracellular lipid was primarily free saturated fatty acid, whereas the cellular lipid droplets contained triglycerides with unsaturated fatty acid chains. Fatty acid uptake and transport were investigated by incubating cartilage samples in Dulbecco's modified Eagle's medium containing fluorescently labelled palmitate for a range of times and temperatures. After incubation, the palmitate distribution was imaged using confocal microscopy. Palmitate accumulated preferentially in the territorial matrix only in the superficial zone where the concentration was up to 100‐fold greater than that in the bulk matrix (*P *= 0.001). Palmitate uptake by the chondrocytes in both zones showed differential temperature sensitivity (*P *= 0.05), which would support the idea that cells take up palmitate by both active and passive mechanisms. The study reveals large differences between chondrocytes in the superficial and deep zones in their lipid content, in their extracellular lipid environment and in their access to exogenous fatty acids.

## Introduction

Lipid metabolism in the chondrocytes of articular cartilage is complex (Villalvilla et al. 2013) and may be involved in the development of disease. There is evidence in humans that changes in the lipid content of cartilage are associated with osteoarthritic (OA) degeneration (Lippiello et al. 1991) and in animal models that manipulation of dietary fatty acids may delay the progression of OA (Lippiello, 1990; Watkins et al. 2001; Curtis et al. 2002), whereas high levels of dietary lipid especially saturated fatty acids increase the rate of joint space narrowing in humans (Lu et al. 2016). An impediment in establishing the mechanisms underlying these observations is that the relationship between dietary lipid availability in the circulation and cellular utilisation is complicated by the behaviour of the lipids in the extensive extracellular matrix, which surrounds every cell. Cartilage is avascular and the extracellular matrix may limit the rate at which lipids can be exchanged with blood, and specific interactions between lipids and the matrix may influence their availability to the cells. A previous study demonstrated such an important role of the extracellular matrix, revealing in particular that fatty acids separate from their natural carrier, albumin, at the cartilage surface and their subsequent behaviour is determined by interactions with the matrix (Arkill & Winlove, 2006).

Extracellular lipid is known to be present in articular cartilage, and the amount in human cartilage has been observed to increase with aging and disease (Stockwell, 1965). Histological staining reveals the majority of extracellular lipid is in the matrix surrounding superficial zone chondrocytes (Bonner et al. 1975). Transmission electron microscopy reveals that this extracellular lipid is in the form of small lipid droplets beneath the diffraction limit for standard light microscopy, with the largest droplets being 0.2 μm in diameter (Ghadially et al. 1965). Some of these droplets show membrane formation, indicating the possible presence of phospholipids (Ghadially et al. 1965). Additionally, lipid droplets within the cells are a common feature within chondrocytes (Collins et al. 1965). Quantitative chemical analyses of cartilage lipids have not distinguished between cellular and extracellular fractions, but have shown that the most abundant fatty acids are oleic (18 : 1), palmitic (16 : 0), linoleic (18 : 2), steric (18 : 0) and arachidonic acid (20 : 4), although both the total quantity and the relative proportions vary with age, species and diet (Adkisson et al. 1991; Lippiello et al. 1991; Xu et al. 1994; Cleland et al. 1995).

The first aim of the present work was to extend our previous work on fatty acid transport to the microscopic scale in order to characterise the three distinct phases of the process: transport through the bulk matrix; interaction with the pericellular matrix; and cellular uptake. The second aim was to relate these processes to the naturally occurring distribution of lipids between these three compartments. Transport of exogenous lipids was investigated using confocal microscopy and fluorescently labelled fatty acids, and the distribution and composition of native lipids were analysed using confocal Raman spectroscopy. Raman spectroscopy relates the spectrum of inelastically scattered light to the bond structure, and hence chemical composition, of the scattering material. It has previously been used to characterise the composition of the cartilage extracellular matrix, with a main focus on the relative changes in collagen and proteoglycan content, and the detection of degeneration or OA (Akira et al. 1994; Bonifacio et al. 2010; Esmonde‐White et al. 2011; Lim et al. 2011; Pudlas et al. 2012; Takahashi et al. 2014; Richardson et al. 2015). Raman spectra have also been obtained from isolated chondrocytes but not, as far as we are aware, from cells embedded within intact tissue (Pudlas et al. 2010, 2012; Kumar et al. 2015). In this study we map the variations in the Raman spectrum across living cells within their native environment, enabling us to investigate differences between chondrocytes from different depths and the relationship between the lipid content of cells and their surrounding matrix.

Both groups of experiments showed perhaps surprisingly large differences between the superficial and deep zones in both cell and matrix lipids in young disease‐free cartilage.

## Experimental

### Cartilage sample preparation

Cartilage samples were taken from bovine metacarpophalangeal joints from animals aged between 24 and 30 months, collected from a local abattoir. The joint was opened under sterile conditions and cartilage plugs were removed from the bone using a scalpel. Prior to imaging they were stored in phosphate‐buffered saline at 4 °C, and all experiments were completed within 36 h of slaughter. Samples from 19 animals were used for the study, with multiple samples from 11 joints used for the Raman analysis and from eight for the fatty acid uptake study.

### Spontaneous Raman scattering

For Raman mapping, thin slices of cartilage (100–200 μm thick) cut parallel to the articular surface were mounted between a glass slide and coverslip, and the edges were sealed with nitrocellulose to avoid dehydration during the imaging process. Raman maps were collected using a WiTec alpha 300r Raman spectrometer with a 532‐nm laser with 34 mW power at the sample and a 63 × 1NA water immersion objective (Zeiss WPlan Apochormat). The Raman maps contained 0.5 μm pixels, and for each pixel the spectral range from 0 to 3800 cm^−1^ was collected, with a spectral resolution of approximately 3.8 cm^−1^. The area of the maps was typically 30 × 30 μm, chosen to cover a chondrocyte and its surrounding matrix. The pixel dwell time was 0.5 s and the spectra were taken at room temperature. The maps were analysed using the witec ‘project four’ software, and fluorescent background was removed using the background subtraction ‘shape function’. To separate out the different components of the cells and extracellular matrix, the maps were analysed using *K*‐means cluster analysis. For each sample, paired slices of superficial zone and deep zone tissue were investigated, and between 1 and 3 cells were mapped in each slice. Polarised light microscopy on bovine cartilage samples revealed that the thickness of the zones was typically: superficial 50–100 μm; transitional 100–200 μm; and deep 600–800 μm. In this study, all superficial zone cells were in the top‐most 0–20 μm and all the deep zone cells were from a depth of greater than 500 μm.

### Fatty acid uptake study

For the uptake measurements, 10 full‐depth cartilage plugs (approximately 1 mm thick and 10 mm^2^ in cross‐section) were cut from the bone in each joint. To aid the visualisation of the chondrocytes, the samples were labelled with the cytoplasmic vital dye CellTracker Red CMTPX (Molecular Probes – Thermo Fisher Scientific) at a 25 μm concentration in Dulbecco's modified Eagle's medium (DMEM) at the start of the study (50 min incubation followed by 30 min washout at 37 °C). Palmitate labelled with BODIPY (Molecular Probes –Thermo Fisher Scientific) was added to DMEM cell culture medium at a concentration of 1 μm with 1% bovine serum albumin. Palmitate was used in the fatty acid uptake study as it is a major component of the extracellular lipid in cartilage. Five of the cartilage plugs were left in the medium containing fluorescently labelled palmitate for incubation times of 20 min, 40 min, 60 min, 4 h and 20 h at 37 °C. To check for irreversible binding, an additional plug was incubated in the labelled medium for 4 h followed by 16 h washout at 37 °C. In order to investigate the temperature dependence of uptake, three additional plugs were incubated for 4 h, 20 h and at 4 h followed by 16 h washout at 4 °C. Lastly, a control plug was incubated in medium without the labelled palmitate. Immediately after incubation, the plugs were placed between two coverslips (giving an axial view of the cartilage, i.e. in the plane parallel to the articular surface), and the palmitate distribution was imaged in both the deep and superficial zones using a confocal microscope (Leica TCS SP5) with a 63 × 1.4NA oil immersion objective. The imaging was performed at room temperature. The uptake at each set time point was measured as the fluorescence intensity in each tissue component of the sample at a depth of between 5 and 10μM below the articular surface. BODIPY fluorescence was excited using a 488‐nm Argon Ion laser, and CellTracker Red was excited with a 594‐nm HeNe laser. The laser powers and PMT settings were kept constant to allow comparison between samples. To measure the partition coefficients and diffusion coefficients, samples were sectioned on a cryo‐microtome after incubation and these sections were imaged using the same imaging parameters. To calculate the diffusion coefficient, we assumed a 1D scenario where the partition coefficient and diffusion coefficient are constant throughout the tissue and there is a constant concentration of palmitate in the bulk solution. In this case, the variation in fluorescence intensity *I* with depth is described by a complementary error function (Crank, 1979):

$$I\left(x,t\right)=I_{0}erfc\left(\frac{x}{2\sqrt{Dt}}\right)$$

where *x* is the distance from the interface with the reservoir, *t* is the incubation time, *I*_0_ is the fluorescence intensity from the reservoir and *D* is the effective diffusion coefficient.

This equation was fitted to the data using matlab. The relative partition coefficients in each zone were calculated from samples that had reached equilibrium concentration, and were based on the relative fluorescence intensity compared with that in the bulk matrix of the deep zone cartilage.

This procedure was repeated on eight joints. The error bars in the results section represent the standard deviation between the measurements from different joints. When comparing between different incubation temperatures and between samples with and without washout, paired *t*‐tests were used comparing paired samples taken from the same bovine joint. When comparing between the deep and superficial zone cartilage, standard *t*‐tests for unequal sample sizes and unequal variances were used.

## Results

### Lipid distributions

**Figure 1:**
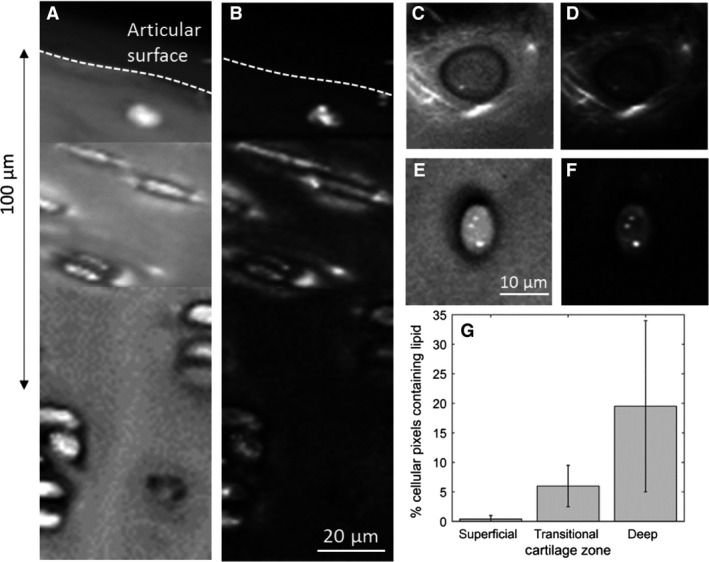
Raman map showing the top 150 μm of a cartilage section cut perpendicular to, and including the articular surface across the CH band (2855–3005 cm^−1^) (A) and across the CH_2_ symmetric stretch band (2840–2850 cm^−1^) (B). (C) and (D) High‐resolution images of a superficial zone chondrocyte mapped across 2855–3005 cm^−1^ and 2840–2850 cm^−1^, respectively. (E) and (F) High‐resolution images of a deep zone chondrocyte mapped across 2855–3005 cm^−1^ and 2840–2850 cm^−1^, respectively. (G) A comparison of fraction of cellular area occupied by lipid droplets in cells of the superficial (sfz), transitional and deep zones.

Spontaneous Raman mapping, like stimulated Raman scattering imaging (Mansfield et al. 2013), allows us to map out the distribution of lipids within cartilage. Figure 1 shows Raman maps, summed over the CH vibration wavelengths 2855–3005 cm^−1^, which includes contributions from CH vibrations in proteins, lipids and proteoglycans giving a good indicator of the amount of organic matter within the samples, and over CH_2_ symmetric stretch vibration 2845–2855 cm^−1^,which represents almost entirely the lipid contribution (Czamara et al. 2015).

From Fig. 1B, it is clear that the distribution of lipids varies with depth into the tissue. Lipid was detected in the matrix close to the cells only in the upper‐most 50 μm immediately below the articular surface. Figure 1D shows a map of the lipid distribution around a superficial zone chondrocyte imaged in the plane parallel to the surface, illustrating a consistent observation that the lipid was distributed in filamentous structures rather than uniformly throughout the matrix. No lipid at all is seen in the matrix surrounding the deep zone cells (Fig. 1F).

Lipids are also present within the chondrocytes, and the amount increases with depth into the tissue. The percentage of pixels identified in the cluster analysis as lipid for the cells in different zones was deep 19.5 ± 14.5 (*n *= 10), transitional 6 ± 3.5 (*n *= 6) and superficial 0.4 ± 0.6 (*n *= 12), as shown in Fig. 1G. The differences between the zones were tested using a *t*‐test (unequal sample sizes, unequal variances) and were all found to be significant (*P *= 0.01).

### Analysis of lipid composition

Mapping the CH_2_ symmetric stretch band provides an indication of the distribution of lipids throughout the tissue, but it does not distinguish the type of lipid present. This requires a more detailed analysis of the whole Raman spectrum.

#### Extracellular matrix

**Figure 2:**
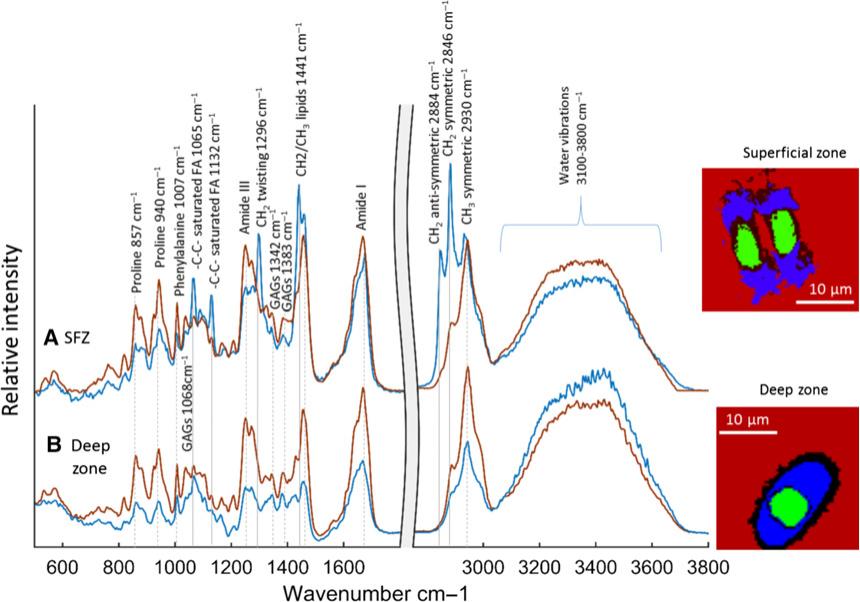
Typical Raman spectra of the territorial matrix (blue) in the deep and superficial zone cartilage compared with spectra from the inter‐territorial matrix away from the cells (red). The spectra have been normalised so the overall area under each spectrum is constant. The spectral region beyond 2700 cm^−1^ has been scaled by 0.25. The peaks that can be attributed to lipid are marked with a solid grey line, whereas the peaks attributed to collagen II and GAGS are marked with a dashed line. The insets show Raman maps of the cells and surrounding matrix from the superficial zone and deep zone (the inter‐territorial matrix is red, the territorial matrix is blue, the cells are green, and transitional regions between cell and territorial matrix and territorial and inter‐territorial matrix are black).

Cluster analysis of the Raman maps clearly distinguished chondrocytes and extracellular matrix. In the extracellular matrix the spectrum was dominated by contributions due to the type II collagen (Bonifacio et al. 2010), with additional peaks attributable to the proteoglycans (Ellis et al. 2009), as summarised in Table 1. In addition, the spectrum of the territorial matrix close to the chondrocytes differed from the bulk inter‐territorial matrix, and was dramatically different between the deep and superficial zones as illustrated in Fig. 2. The peak attributions in each of these tissue components are summarised in Table 1.

**Table 1:** The Raman peaks identified in different cartilage regions and their assignments

Peak cm^−1^	Assignment	Ref.	Lipid droplets	Cells	Inter‐terrritorial matrix	Territorial matrix deep	Territorial matrix sfz
3005	=C‐H stretching	(Czamara et al. 2015)	w	–	–	–	–
2930	CH_3_ symmetric stretch	(Czamara et al. 2015)	s	s	s	s	s
2980	CH_2_ anti‐symmetric stretch	(Czamara et al. 2015)	s	m	m	m	vs
2850	CH_2_ symmetric stretch (unsaturated)	(Czamara et al. 2015)	s	w	–	–	–
2845	CH_2_ symmetric stretch (saturated)	(Czamara et al. 2015)	–	–	–	–	s
1750	Ester bond	(Czamara et al. 2015)	w	–	–	–	–
1670	Amide I	(Bonifacio et al. 2010)	–	s	s	s	s
1660	‐C=C‐ unsaturated lipids	(Czamara et al. 2015)	s	–	–	–	–
1457	CH protein		–	s	s	s	s
1441	CH lipids	(Czamara et al. 2015)	s	–	–	–	–
1383	GAGS	(Ellis et al. 2009; Bonifacio et al. 2010)	–	vw	w	m	w
1342	GAGS	(Ellis et al. 2009; Bonifacio et al. 2010)	–	s	w	m	w
1305	CH_2_ twisting (unsaturated lipids)	(Czamara et al. 2015)	s	–	–	–	–
1296	CH_2_ twisting (saturated lipids)	(Czamara et al. 2015)	–	–	–	–	s
1265	=C‐H	(Czamara et al. 2015)	s	–	–	–	–
1269 (shoulder)	Amide III	(Bonifacio et al. 2010)	–	s	s	s	m
1245	Amide III	(Bonifacio et al. 2010)	–	s	s	s	s
1132	‐C‐C‐ skeletal sat fatty acid	(Czamara et al. 2015)	–	–	–	–	m
1084–7	‐C‐C‐ skeletal unsaturated fatty acid	(Czamara et al. 2015)	m	–	–	–	–
1068	GAGS	(Ellis et al. 2009; Bonifacio et al. 2010)	–	–	w	m	–
1065	‐C‐C‐ skeletal sat fatty acid	(Czamara et al. 2015)	–	–	–	–	m
1007	Phenylalanine	(Bonifacio et al. 2010)	w	m	m	m	m
940	Proline	(Bonifacio et al. 2010)	–	m	s	m	m
920	Proline/ hydroxyproline	(Bonifacio et al. 2010)	–	m	m	m	m
876	Proline/ hydroxyproline	(Bonifacio et al. 2010)	–	m	m	m	m
857	Proline	(Bonifacio et al. 2010)	–	m	s	m	m

vs, very strong; s, strong; m, medium; w, weak; vw, very weak.

**Figure 3:**
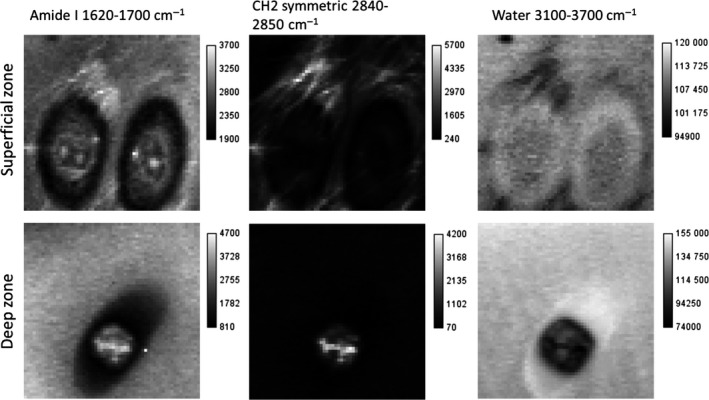
Raman maps of chondrocytes in the superficial and deep zones (amide I – intensity sum 1600–1720 cm^−1^, lipid intensity sum 2845–2855 cm^−1^ and water intensity sum 3100–3800 cm^−1^; all images 30 × 30 μm).

As noted above, lipids were evident in the territorial matrix of the superficial zone chondrocytes. The spectra showed peaks at 1065 cm^−1^ and 1132 cm^−1^ (skeletal ‐C‐C‐ stretching) and 1296 cm^−1^ (CH_2_ twisting; Czamara et al. 2015), and in the CH vibrational region between 2800 and 3000 cm^−1^ two additional strong peaks at 2846 cm^−1^, 2884 cm^−1^, which are due to CH_2_ symmetric and antisymmetric stretching. These modes are characteristic of the long‐chain alkanes in saturated fatty acids (Czamara et al. 2015). Furthermore, the spectra contained no ester peak at about 1750 cm^−1^, indicating that the lipid is in the form of free fatty acids. In these lipid‐rich regions there were still strong spectral contributions from protein and proteoglycans. Subtle differences in the shape of the amide I peak were observed compared with the neighbouring lipid‐free matrix, with a narrow peak at 1683 cm^−1^ with a shoulder at 1663 cm^−1^ in the lipid‐rich matrix compared with a peak at 1670 cm^−1^ with a shoulder at about 1635 cm^−1^ in the inter‐territorial matrix. The amide III band shape was also different. These differences suggest alterations in collagen structure or changes in protein composition. Additionally, the height of the proline peaks at 857 cm^−1^ and 940 cm^−1^ was reduced compared with the amide I peak in the lipid‐rich regions. As proline is a major component of collagen, the relative reduction in the height of the proline peaks indicates a lower collagen content compared with the inter‐territorial matrix. In the lipid‐rich regions, the amplitude of the water peak decreased relative to amide I, suggesting that water is excluded from the lipid‐rich regions (Fig. 3).

These spectral features were observed in the territorial matrix of 94% of the superficial zone chondrocytes investigated. However, these features were completely absent from all of the deep zone samples. In the deep zone of articular cartilage, the main spectral differences between the territorial matrix and the bulk were in the amplitudes of the glycosaminoglycan and water peaks relative to amide I, indicating an increased proportion of glycosaminoglycans and water close to the cells.

#### Chondrocytes

**Figure 4:**
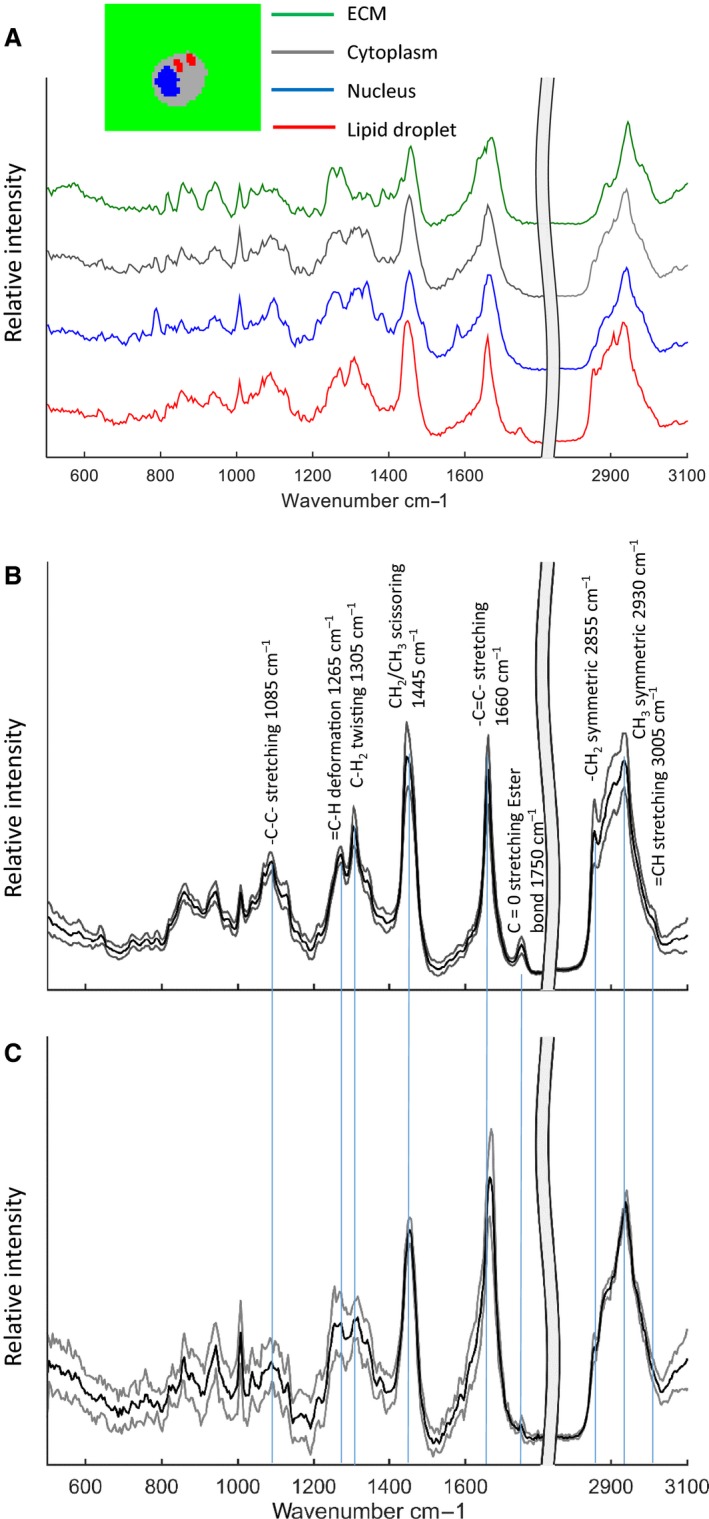
Cluster analysis results from a deep zone chondrocyte, showing four clusters that can be identified as the extracellular matrix (ECM), cell cytoplasm, nucleus and lipid droplet. Average lipid droplet spectra from deep and transitional zones (B) and superficial (C) (the spectral region beyond 2700 cm^−1^ has been scaled by 0.25) grey lines are average spectra ± 1 standard deviation [*n *= 9 for the deep zone (B) and *n *= 5 for the superficial zone (C)].

The lipid droplets present in many of the chondrocytes could readily be distinguished from the other cellular components in the Raman maps by *K* means cluster analysis (Fig. 4A). The spectra of the cellular lipid droplets (Fig. 4B and C) were very different to those of the lipid‐rich regions in the extracellular matrix. Most notably there was evidence of esterification (1750 cm^−1^ peak) and unsaturation (peaks at 1265 cm^−1^ and 1660 cm^−1^ and a shoulder at 3005 cm^−1^), supported by the CH_2_ twisting peak at 1305 cm^−1^ and the broad ‐C‐C‐ skeletal mode at 1084 cm^−1^. Together, these features are characteristic of unsaturated triglycerides at room temperature. The ratio of the 1655 cm^−1^ ‐C=C‐ peak and the 1445 cm^−1^ for CH_2_ scissoring has been used as a linear measure of the average number of C=C bonds per molecule (Czamara et al. 2015). This ratio ranged from 0.87 to 1.21 in our samples, corresponding to between 1.43 and 2 double bonds per fatty acid molecule.

### Transport and uptake of fatty acid in cartilage

**Figure 5:**
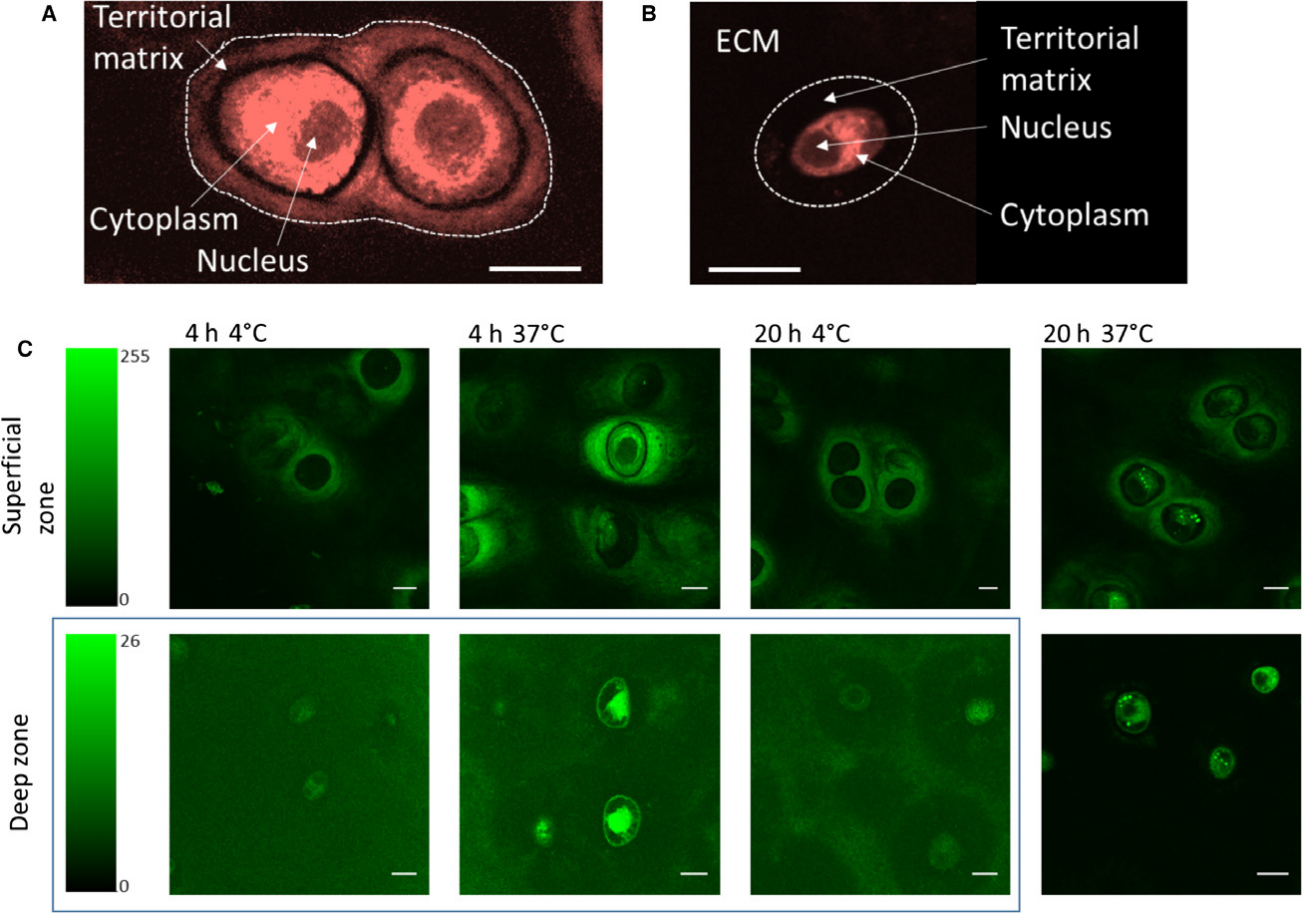
(A) and (B) Confocal images of chondrocytes labelled with cell tracker red from the superficial zone and deep zone, respectively. (C) Images of labelled palmitate in chondrocytes after different incubation times (Scale bars: 10 μm).

The transport and cellular uptake of fluorescently labelled palmitate was measured in both the superficial and deep zones of cartilage plugs. Fluorescence intensity in the extracellular matrix, territorial matrix, cytoplasm and nucleus were measured after incubation times of between 0 and 20 h. Figure 5A and B shows how these regions were defined relative to cell boundaries delineated by the CellTracker Red label. For the calculation of cellular uptake, only cells that had labelled positively with the Cell‐Tracker Red and showed an intact nucleus were used in the analysis, thus excluding dead cells. Typical images showing the distribution of labelled palmitate in cells and their surrounding matrix are shown in Fig. 5C.

**Figure 6:**
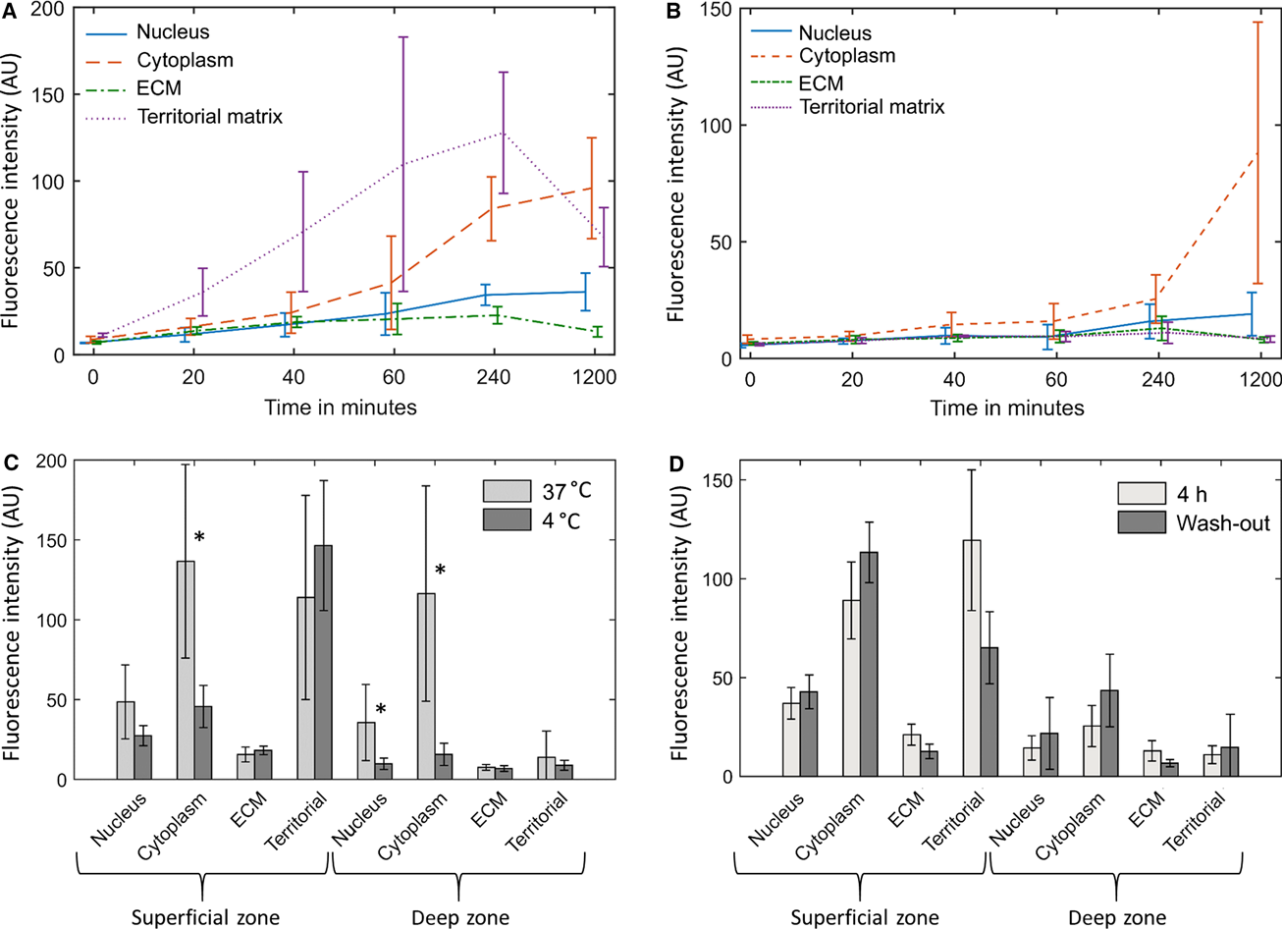
Time‐course for the uptake of palmitate in both the superficial and deep zones (A and B, respectively). (C) Relative amounts of palmitate fluorescence in different components of the cartilage after 20 h incubation in labelled medium at 4 and 37 °C. (D) Relative amounts of palmitate fluorescence in different components of cartilage following 16 h washout period after 4 h incubation time compared with 4 h incubation and no washout (both at 37 °C). The error bars represent the standard deviation between all the samples investigated (*differences that are significant at the *P *= 0.05 level; for A and B, *n *= 4; for C and D, *n *= 6).

The time course of palmitate uptake is shown for the superficial and deep zones in Fig. 6A and B, respectively. Figure 6A shows that over the time period investigated, palmitate preferentially accumulated in the territorial matrix of the superficial zone chondrocytes. In the deep zone matrix, palmitate did not accumulate to a measurable extent in the territorial matrix [no statistical difference (*t*‐test, *P *= 0.05 significance level) from the control samples for all incubation times]. The chondrocytes in both the superficial and deep zones took up the labelled palmitate. Over the time period of 0–4 h, the rate of uptake was roughly linear in both zones, but the rate was approximately 4.5 times faster in the superficial zone chondrocytes than in the deep zone. However, it is impossible to establish whether these differences are determined by differences in cellular lipid uptake kinetics or in the rates of transport in the superficial and deep zone matrix.

To establish whether the cellular uptake of palmitate was an active or passive process, samples incubated in labelled palmitate at 4 and 37 °C for 20 h were compared (Fig. 6C). Paired *t*‐tests were applied to the data for each temperature group, and the hypothesis that there was no difference between the two groups was tested at the *P *= 0.05 significance level. For the inter‐territorial and territorial matrix, differences in concentration were not statistically significant. However, the amount of palmitate in the cytoplasm was significantly lower at 4 °C for both the deep and superficial zone chondrocytes (15 ± 8% and 35 ± 17% of the total at 37 °C for the deep and superficial zones, respectively). This indicates that at least one of the rate‐determining steps is likely to be an active process, although significant uptake still occurred at 4 °C, indicating a basal level of passive diffusion.

To investigate palmitate binding, paired tissue samples were compared immediately after 4 h incubation and 4 h incubation followed by 16 h washout in unlabelled medium, as shown in Fig. 6D. The amount of palmitate in the matrix decreased after washout to 56 ± 15% (*n *= 6) of the value for 4 h incubation. However, the cytoplasmic concentration continued to increase during the washout period by between 27% and 72%, providing further evidence of an active cellular uptake mechanism.

**Figure 7:**
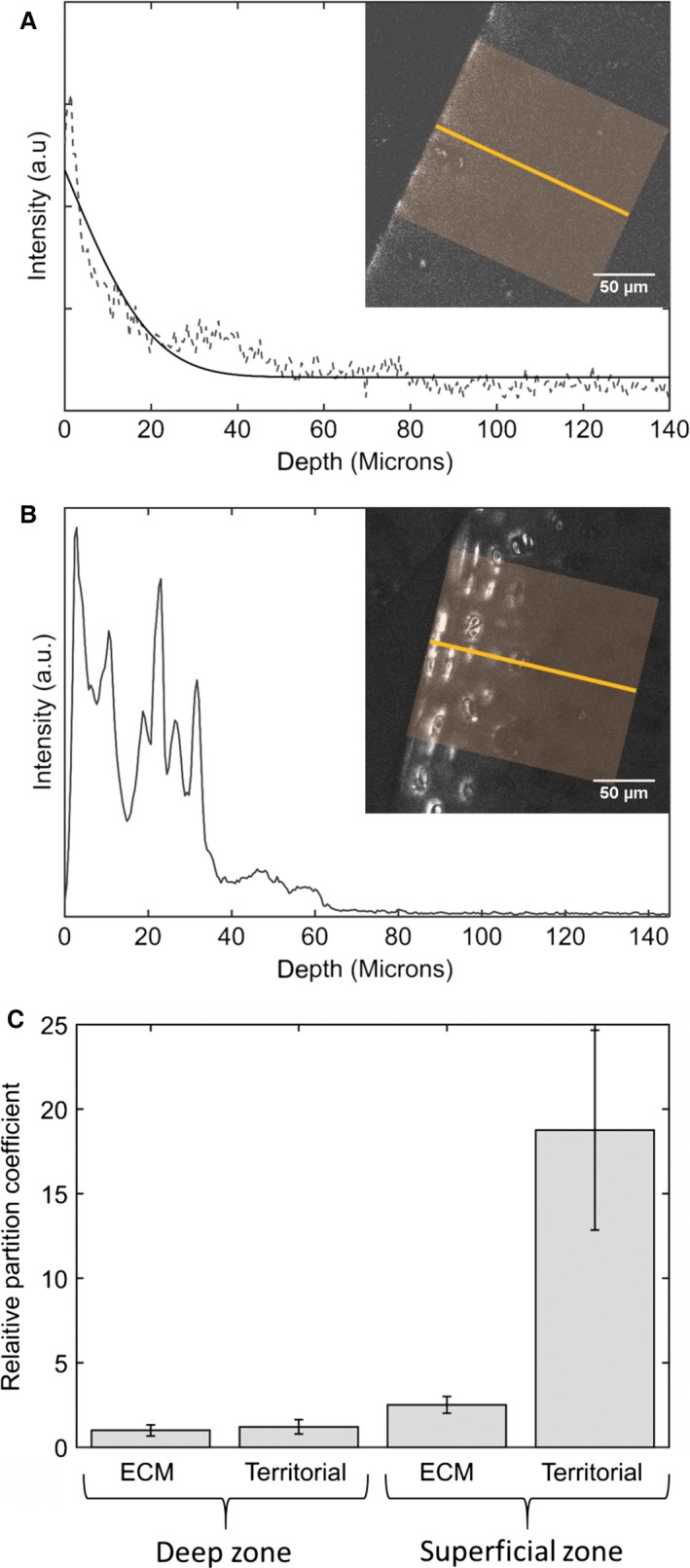
(A) and (B) Plots of intensity vs. depth in histological sections taken from the deep and superficial zones, respectively. A complementary error function is fitted to the data in (A) allowing the effective diffusion coefficient to be estimated; however, for (B) the data were too heterogeneous to fit. (C) Relative partition coefficients in the deep and superficial zone matrix calculated after 18 h incubation at 4 °C (mean ± SD,* n *= 6).

The transport of palmitate in cartilage is dependent upon the diffusion coefficient and the partition coefficient of the palmitate in the extracellular matrix. The effective diffusion coefficient was estimated from the plots of the intensity vs. depth of histological sections with known incubation times (Fig. 7A and B). Fitting a complementary error function to the plots of tracer concentration with depth gave an estimate of the effective diffusion coefficients within a range of 1–3.6 × 10^−14^ m^2^s^−1^ for the deep zone. This value is two orders of magnitude smaller than the diffusion coefficient for a smaller lipid lauric acid measured by Arkill & Winlove (2006), which may indicate differences in hydrophobicity and interaction with matrix molecules or specific carrier molecules. It was not possible to determine an effective diffusion coefficient in the surface zone because both the pathways and interactions with the matrix were so heterogeneous as to generate extremely noisy concentration profiles (as shown in Fig. 7B). The partition coefficients were much greater in the superficial zone compared with the deep zone (*t*‐test unequal sample size unequal variance *P *= 0.001; Fig. 7C). In addition, whilst the partition coefficient was relatively homogeneous throughout the deep zone matrix in the superficial zone, there were large differences between the partition coefficient between the bulk and the territorial matrix with a much higher partition coefficient in the territorial matrix (*t*‐test unequal sample size unequal variance *P *= 0.001).

## Discussion

Our results raise several questions about the extracellular lipid in the superficial zone. Firstly, why do lipids preferentially accumulate in the matrix surrounding the superficial zone chondrocytes? The Raman spectra of these regions indicated that they contained less collagen than the rest of the matrix, and also contained some proteins with different secondary structures. Potentially the different proteins could provide a more favourable environment for lipids. Secondly, where does the extracellular lipid come from: is it generated by the cells or filtered out of the synovial fluid? At the articular surface a boundary layer of lipids is reported and its composition was analysed by Sarma et al. (2001), showing that the lipids were phosphatidylcholine (41%), phosphatidylethanolamine (27%) and sphingomyelin (32%) with oleic acid as the most abundant fatty acid. Phosphatidylethanolamine and sphingomyelin have peaks at 719 cm^−1^, 760 cm^−1^ and 723 cm^−1^, respectively and also a peak at 1098 cm^−1^ corresponding to a phosphate group (Czamara et al. 2015), which was absent in our spectra of the lipid‐rich regions surrounding the chondrocytes. This indicates that these lipids are not the same composition as those found in the boundary layer by Surma et al. Furthermore, Raman spectra of the synovial fluid extracted from the joints used in this study showed no lipid peaks, and were dominated by protein peaks and carotenoid peaks (data not shown), indicating that the lipid concentration in synovial fluid is very low and is therefore perhaps unlikely to be the principal source of tissue lipids. An alternative theory is that the extracellular lipid is debris from dead cells. However, this also appears unlikely as the lipid was consistently found surrounding viable cells in young healthy tissue and, in addition, the lipid composition is different to that within the cells. We therefore suggest that the lipids are externalised from living cells or the cells are selective in their uptake of fatty acid.

The intracellular lipid droplets provide evidence of active lipid processing. The lipid droplets within the chondrocytes contained mostly unsaturated fatty acids in triglycerides. The size of the lipid droplets was found to increase with depth into the cartilage, but we were unable to compare the composition of the lipid droplets with depth as those in the superficial zone are too small to provide reliable spectra. This study focused on cartilage from young healthy bovines, and an obvious extension of this work would be to investigate differences in lipid concentration between different age groups and diets, and to extend this to investigating the composition of the lipids in human articular chondrocytes. Additionally, it would be interesting to investigate if there is any link between cellular lipid content and nucleic acid content and markers for metabolic activity.

The effective diffusion coefficients of palmitate in the extracellular matrix were similar between the deep and superficial zones; however, the two zones showed very different partition coefficients, which were much greater and more heterogeneous in the superficial zone matrix. Fatty acid accumulates in the territorial matrix of the superficial zone chondrocytes and this is accentuated by strong binding to the matrix, as 50–60% of the fatty acid remains in the matrix after extensive washing. One limitation of our study is that the diffusion measurements were carried out on cartilage that had been removed from the bone, to do this the deep zone cartilage had to be cut, which may have increased the permeability of the tissues, therefore in physiological conditions the rate of uptake and diffusion coefficient for the deep zone may be lower. Additionally, it is also important to note that palmitate and BODIPY are similar sized molecules, and therefore tagging the palmitate with the BODIPY molecule will potentially alter its transport properties. A potential way to overcome this problem in future studies would be to investigate the uptake of deuterium‐labelled palmitate using coherent Raman techniques (Belsey et al. 2014). All the transport measurements reported in this study were on unloaded cartilage; however, *in vivo* the cartilage would be undergoing cyclic compressive loading associated with joint movements. As palmitate has a low diffusion coefficient within cartilage its transport is likely to be affected by the increased convection caused by cyclic loading (O'Hara et al. 1990), and investigating these effects would be an important extension of this study.

The uptake of palmitate into the chondrocytes showed differential temperature sensitivity, which supports a hypothesis that the chondrocytes accumulate palmitate by both active and passive mechanisms. Further work with inhibitors of specific transport pathways could clarify the precise mechanisms. Such work might also determine whether the higher rate we observed in the superficial zone compared with the deep zone is due to a physiological difference in the chondrocytes in the different zones or because the extracellular matrix in the superficial zone provides a reservoir for fatty acids.

This study has clearly shown that the superficial zone and deep zone chondrocytes have a very different environment in terms of both the amount of lipid in the matrix surrounding the cells and the availability of lipids via diffusion though the matrix. Because the availability of lipids has been shown to affect the metabolism of chondrocytes, it could contribute to the cellular heterogeneity in normal cartilage, and because the amount of lipid and the rate of lipid diffusion in cartilage increase in OA cartilage it may also be an important factor in the development of disease.

The present study demonstrated that territorial matrix environment is very different between the deep and superficial zones. The highly hydrated proteoglycan‐rich matrix in the deep zone and the lipid‐rich matrix surrounding the superficial zone cells may provide very different barriers to the exchange of hydrophobic or hydrophilic signal molecules, and one can speculate that they may have very different mechanical properties that would affect the transmission of mechanical forces to the cells. These regional differences should perhaps be borne in mind when such processes are studied in chondrocytes separated from their native environment.

## Author contributions

JCM was responsible for the experimental work and data analysis. Both authors worked together on the design of the study, interpretation of the results and editing of the manuscript.
